# Study protocol: development and randomized controlled trial of a preventive blended care parenting intervention for parents with PTSD

**DOI:** 10.1186/s12888-023-04548-8

**Published:** 2023-02-10

**Authors:** Laurien Meijer, Catrin Finkenauer, Matthijs Blankers, Anouk de Gee, Jeannet Kramer, Laura Shields-Zeeman, Kathleen Thomaes

**Affiliations:** 1 Sinai Centrum / Arkin Mental Health Care, Laan van de Helende Meesters 2, 1186 AM Amstelveen, The Netherlands; 2 Department of Interdisciplinary Social Science, Utrecht University, Utrecht, The Netherlands; 3 Arkin Mental Health Care, Amsterdam, the Netherlands; 4 Trimbos Institute/Netherlands Institute of Mental Health and Addiction, Utrecht, the Netherlands; 5 Department of Psychiatry, Amsterdam University Medical Center, location VUmc, Amsterdam, the Netherlands

**Keywords:** Post-traumatic stress disorder, Trauma, Parenting, Children of parents with mental illness, Intergenerational transmission of trauma, Preventive intervention, Randomized controlled trial, Study protocol

## Abstract

**Background:**

Children of parents with post-traumatic stress disorder (PTSD) are at increased risk of adverse psychological outcomes. An important risk mechanism is impaired parental functioning, including negative parenting behavior, perceived incompetence, and lack of social support. Several parenting interventions for trauma-exposed parents and parents with psychiatric disorders exist, but none have specifically targeted parents with PTSD. Our objective is to evaluate the effectiveness of a blended care preventive parenting intervention for parents with PTSD.

**Methods:**

The intervention was adapted from an existing online intervention, KopOpOuders Self-Help. In co-creation with parents with PTSD and partners, the intervention was adapted into KopOpOuders-PTSD, by adding PTSD-specific content and three in-person-sessions with a mental health prevention professional. Effectiveness will be tested in a randomized controlled trial among *N* = 142 parents being treated for PTSD at Arkin Mental Health Care (control condition: treatment as usual, *n* = 71; intervention condition: treatment as usual + intervention, *n* = 71). Online questionnaires at pretest, posttest, and three-month follow-up and ecological momentary assessment at pretest and posttest will be used. Intervention effects on primary (parenting behavior) and secondary outcomes (perceived parenting competence, parental social support, parenting stress, child overall psychological problems and PTSD symptoms) will be analyzed using generalized linear mixed modeling. We will also analyze possible moderation effects of parental PTSD symptoms at pretest on primary and secondary outcomes.

**Discussion:**

This study protocol describes the randomized controlled trial of KopOpOuders-PTSD, a blended care preventive parenting intervention for parents with PTSD. Findings can contribute to understanding of the effectiveness of parenting support in clinical practice for PTSD.

**Trial registration:**

This protocol (Version 1) was registered on 11-02-2022 at ClinicalTrials.gov under identification number NCT05237999.

**Supplementary Information:**

The online version contains supplementary material available at 10.1186/s12888-023-04548-8.

## Background

Post-traumatic stress disorder (PTSD) is a psychological disorder which follows one or more traumatic experiences and is characterized by four symptom clusters: intrusion (e.g., intrusive mental images or sensations related to the trauma), avoidance (e.g., avoiding stimuli that may serve as reminders of the trauma), negative alterations in cognitions and mood (e.g., feelings of shame and guilt), and increased arousal and reactivity (e.g., hypervigilance and irritability) [1]. These symptoms can disrupt individual wellbeing, as well as interpersonal functioning (e.g., work, relationships, family life) [1]. Although many people experience one or more potentially traumatic events during their life, only a small percentage develops PTSD. Using criteria from the Diagnostic and Statistical Manual of Mental Disorders-Fifth Edition (DSM-5 [1]), 43.8% of a Dutch sample had been exposed to one or more potentially traumatic events during their lifetime, whereas past-week PTSD prevalence was 2% [2]). Rates of trauma exposure and PTSD prevalence often vary between countries. This can be due to methodological differences between studies, but also to factors such as war and social inequality [2]. For instance, using the same DSM-5 criteria in a United States sample, lifetime trauma exposure prevalence was 89.7% and lifetime PTSD prevalence 8.3% [3].

Many adults living with PTSD are parents [4]. Parental PTSD is associated with a range of adverse child psychological outcomes. These outcomes include PTSD as well as anxiety, depression, general psychological distress and externalizing behavior, even in instances when the child was not exposed to the traumatic event(s) preceding parental PTSD [5]. Targeting this intergenerational transmission process can be an important route for preventing mental illness in children. Pathways between parental PTSD and child psychological health are complex and include biological (e.g., epigenetic processes), environmental (e.g., societal inequality, adverse community environments) and relational (e.g., parent-child interaction) mechanisms [6]. Relational mechanisms can provide especially promising avenues for preventive intervention, because they can be more directly accessed and influenced than biological and environmental mechanisms [6]. Parenting and the parent-child relationship are therefore relatively popular intervention targets for parents with mental illness.

### Existing parenting interventions for mental illness and trauma

Meta-analytical evidence shows that preventive parenting interventions for parents with mental illness (mostly aimed at parents with mood and substance use disorders) have generally small, but consistent positive effects on child psychological health [7, 8]. Some interventions for trauma-exposed parents, not specifically with PTSD, have also been developed and studied. These interventions target specific trauma populations, for example, survivors of domestic violence [9–12], survivors of childhood trauma [13, 14], people with refugee backgrounds [15, 16], and veterans [17, 18]. While keeping variations in intervention content, populations and methodology across studies in mind, it can be broadly concluded that positive effects are found in parents and children. Effects pertain particularly to perceived parenting competence and child psychological health, especially externalizing symptoms such as hyperactivity and behavior problems [9–18]. Recently, a small-scale pilot study of a trauma-informed parenting intervention for veterans with PTSD or elevated PTSD symptoms found promising results, with findings suggesting improvements on family functioning and parenting behavior [19]. To our knowledge, this is the first parenting intervention that specifically targets parents with PTSD.

Although most parenting interventions for parents with mental illness are in-person, a small number of online interventions also exists. These online parenting interventions for parents with mental illness have targeted parents with bipolar disorder [20], mothers with depressive, bipolar, or schizophrenia spectrum disorders [21], or parents with all types of mental illness and/or addiction [22–24]. Although trauma exposure and PTSD are not exclusion criteria, none of these interventions have specifically targeted trauma-exposed parents or parents with PTSD. One pilot study included military veteran parents [25]. Whereas having mental illness or experiencing trauma was not an inclusion criterion, many participants were receiving mental healthcare through Veterans Affairs and trauma exposure is very common in this population. Therefore, we will also consider the results of this study here. Positive effects of online interventions are mainly found on parenting behavior and perceived parenting competence, with significant improvements on both domains [20, 22–24]. Parental social support was an outcome variable in some studies, but did not increase significantly [21, 22, 24]. Parenting stress was measured in only two studies, where it decreased significantly [20, 25]. Significant improvements in child psychological health were found in most online interventions [20, 22, 24, 25]. Studies that distinguished between mental health domains found the strongest effects on hyperactivity and emotional problems [22, 24].

### PTSD-specific parenting intervention

Although positive effects of existing interventions have been found in trauma-exposed parents and parents with mental illness, there are several reasons why these may not always translate to parents with PTSD. Firstly, PTSD symptoms are known to interfere with intervention adherence and effectiveness in a wide range of interventions [26–28]. PTSD symptoms such as reduced cognitive and memory functioning and avoidance of potentially triggering stimuli are thought to underlie this issue [27, 28]. To our knowledge, only one study has tested whether PTSD symptoms interfere with parenting intervention effectiveness. This study found lower effectiveness of a parenting intervention in veteran fathers (but not mothers; although it must be noted that the number of mothers with PTSD was very small) who had PTSD than in those who did not [29]. Furthermore, PTSD symptoms can evoke specific parenting challenges (e.g., dissociation or aggressive outbursts in the presence of children in response to intrusions or ‘flashbacks’; inability to participate in family activities due to avoidance of triggers), which are not covered in other parenting interventions [30, 31].

Additionally, although existing parenting interventions for trauma-exposed parents are all targeted towards specific trauma types, there might actually be more commonalities than differences between parents with PTSD resulting from different trauma types. Several studies have tested the effects of number of parental traumas and parental PTSD symptoms on parenting [32–35]. Adaptive dimensions of parenting (e.g., involvement, satisfaction, autonomy support) appear to be impacted by PTSD rather than number of traumatic experiences. One study showed that any links between number of traumatic experiences and parenting outcomes (involvement, appropriate limit setting, autonomy granting) were fully mediated by PTSD symptom severity [32]. In a different study, the number of traumatic experiences was not significantly associated with any parenting outcome (parenting behavior, perceptions, support, or attitudes), regardless of whether PTSD symptoms were controlled for [33]. Findings are more mixed for maladaptive dimensions of parenting (e.g., harshness). One study found significant effects of number of traumatic experiences, not PTSD diagnostic status, on harsh and abusive parenting [34]. Another found both number of traumatic experiences and PTSD symptom severity to predict child abuse potential [35].

More information on the relative contributions of trauma and PTSD to parenting challenges is needed, especially in clinical samples and focusing on the type, not just number, of traumatic experiences. A systematic review found higher risk of child abuse perpetration in parents with PTSD compared to trauma-exposed parents without PTSD, regardless of trauma type [36]. Studies that explicitly test the effect of trauma type on non-abusive parenting dimensions in parents with PTSD are rare; to our knowledge, only one study has tested this, but the small sample size impedes drawing robust conclusions [37].

In short, there is currently not enough consistent empirical evidence to conclude whether trauma type significantly impacts parenting in parents with PTSD. As such, we consider it sensible to offer a parenting intervention to all parents with PTSD, regardless of the type of trauma they have experienced.

Finally, the current range of parenting interventions for trauma-exposed parents has addressed many trauma types, but (to the best of our knowledge) has not included some relatively common types of trauma exposure, such as disasters and accidents [3]. A secondary advantage of PTSD-specific parenting intervention that caters to parents regardless of trauma type is that it helps partially close this gap.

### KopOpOuders-PTSD

These arguments have inspired the development of KopOpOuders-PTSD, a parenting intervention specifically for parents with PTSD. KopOpOuders-PTSD is meant to be completed in addition to PTSD treatment and is delivered in a blended care format, which means it combines online modules with in-person sessions.

#### KopOpOuders self-help

KopOpOuders-PTSD is an adaptation of the existing intervention ‘KopOpOuders Self-Help’ [22]. ‘KopOpOuders’ means ‘Chin up, parents’ and is a play on words on the Dutch acronym ‘KOPP’ (children of parents with mental illness). KopOpOuders Self-Help is used as the basis for our PTSD-specific intervention, because it is the most long-standing and widely used online preventive parenting intervention in the Netherlands. KopOpOuders Self-Help caters to parents with mental illness or addiction, regardless of whether they have received a diagnosis or are in treatment. KopOpOuders Self-Help strives to enhance parental autonomy and self-direction. This is expressed in both content (e.g., encouraging parents to activate their own social network) and form (e.g., homework exercises, minimal contact with a professional [38]). The effectiveness of KopOpOuders Self-Help has been tested in two pilot studies [22, 24]. Participants were parents of children aged 0–18, had any type of mental illness or addiction, and had signed themselves up (resp. *N* = 33 and *N* = 42). A pretest-posttest design was used in which participants completed online questionnaires before starting and after completing the course. Significant improvements from pretest to posttest in parenting behavior (total score, overreactivity and permissiveness) were found [22]. Perceived parenting competence increased significantly in both studies [22, 24]. Child psychological wellbeing (reported by the parent) also increased, with significant improvements on total problems, emotional problems and hyperactivity and total problems [22, 24].

#### Theoretical framework

KopOpOuders-PTSD is built on the same theoretical framework as KopOpOuders Self-Help. This framework is also the same as that of Triple P, a proven effective parenting intervention [39, 40]. It is based on insights from social cognitive theory [41, 42], the social processing model [41, 43], developmental psychology [44], developmental psychopathology [45, 46], and the contextual approach [47].

#### Social cognitive theory

Social cognitive theory [41, 42] provides a framework for recognizing learning processes in parenting and parent-child interactions. This is operationalized by teaching parents that their child learns from observing their behavior. Patterson’s extension of social cognitive theory [42] is applied in teaching parents how children and parents influence each other. Parents are made aware of coercive interaction patterns and learn to interrupt them. Additionally, they learn to promote desirable behavior through reinforcement [38].

#### Social processing model

The social processing model [41, 43] stresses the social-cognitive processes influencing parents’ behavior. Negative thought patterns (e.g., hostile attributions) can adversely impact parent-child interactions [48]. In KopOpOuders-PTSD, parents are encouraged to identify and recognize such negative attributions. Negative self-attributions (e.g., shame and guilt about the family situation, perceived parenting incompetence) are also identified and reduced [38].

#### Developmental psychology

Insights from developmental psychology [44] are used to teach parents about the normal and expected behaviors at each developmental stage of their child, and developmentally appropriate ways to inform the child about the parent’s mental illness [38].

#### Developmental psychopathology

Insights from the field of developmental psychopathology [45, 46] provide a framework of known risk and protective factors for development of children growing up with a parent with mental illness (parental self-perceptions, parent-child interaction quality, social support, child adaptive functioning/coping, and child understanding of the parent’s illness [49–52]). Parents learn about these factors through psychoeducation and are supported in reinforcing protective factors [51].

#### Contextual approach

The contextual approach [47] emphasizes the importance of the informal support network (family and everyday social contacts) for individual functioning and recovery. In KopOpOuders-PTSD, parents are supported in expanding or strengthening the social network, and by involving the partner or other significant person [38].

To summarize, the KopOpOuders-PTSD framework includes behavioral, cognitive and social elements. From this framework, three main targets of the intervention arise: parenting behavior, perceived parenting competence, and parental social support. Additionally, KopOpOuders-PTSD is hypothesized to reduce parenting stress, child psychological problems and child PTSD symptoms. It is important to note that the effects of PTSD on parenting are complex, and that KopOpOuders-PTSD does not address every aspect of parenting that might be affected by PTSD. For example, parental PTSD can influence parent-child attachment (e.g. [53, 54]) and this can also impact parent-child bonding and child wellbeing later in life [54, 55]. However, because KopOpOuders-PTSD is meant as preventive, short-term and accessible, these more complex relational mechanisms are not specifically addressed. They are therefore not further discussed in this paper.

### Parenting behavior

A range of studies shows that PTSD symptoms are associated with negative parenting behaviors. These include over-reactive behaviors, such as harshness, intrusiveness and overcontrol, as well as under-reactive behaviors, such as permissiveness, poor supervision, and withdrawal [56–59]. Furthermore, parents with PTSD are more likely than trauma-exposed parents without PTSD to perpetrate child maltreatment and neglect [36]. Whereas most of the literature finds PTSD symptoms to be related to more negative parenting behaviors [56], it is important to note that this association is not found in all studies (e.g., [54, 60]) and that as of yet, little is known about factors that make it more or less likely for parenting to be affected by PTSD. Furthermore, PTSD symptoms do not appear to be related to less positive parenting behaviors [56]. Thus, learning how to recognize negative parenting behaviors and replacing them with more positive alternatives may be especially important for parents with PTSD.

### Perceived parenting competence

On average, parents with PTSD consider themselves less competent as parents [56, 59]. Although there may be actual deficits in parenting behavior, negative self-cognitions and cognitive biases present in PTSD are also thought to influence perceived parenting competence, resulting in overly negative views of one’s own parenting competence [33, 61]. This negative bias is further reflected in findings showing limited convergence between observed and self-reported parenting in parents with traumatic experiences and/or PTSD, with parents reporting on themselves more negatively than observed [60, 62–64]. Depression, which is highly comorbid with PTSD and has considerable symptom overlap with the PTSD symptom cluster of negative alterations in cognitions and mood, appears to play an explanatory role in this perceived parenting incompetence [60, 62, 63, 65]. Apart from parents’ well-being, perceived parenting incompetence can also negatively impact parenting behavior. In trauma-exposed parents, negative parenting perceptions were found to mediate the relationship between PTSD symptoms (specifically those in the arousal and reactivity cluster) and parental involvement [61]. This is in line with the broader parenting literature, which shows higher perceived parenting competence to contribute to more positive and less negative parenting behavior [66]. Furthermore, perceived parenting competence has been found to mediate effects of preventive parenting interventions on adaptive parenting behavior [67] and parental stress [68].

Because of the potential negative impact of perceived parenting incompetence on parents’ wellbeing and its effects on parenting behavior, we consider it beneficial to promote perceived parenting competence in parents with PTSD.

### Social support

PTSD is associated with erosion of social support (defined as “actual or available social resources in times of need and groups involved that are perceived as positively supportive” [69] (p.2)). This can be harmful not only for parents with PTSD but also for their children, as lack of parental social support can also contribute to adverse child outcomes. Availability of social support resources to parents can mitigate negative effects of parental mental illness on children [70–73]. Although there is little research so far about the importance of parental social support for children of parents with PTSD specifically, its role as a buffer against intergenerational risk transmission following parental trauma exposure more generally is well-supported in the literature. For example, in mothers who had experienced childhood trauma, receiving more social support was found to diminish risk of future child maltreatment perpetration [70] and mother-child transmission of biological risk (i.e., cortisol reactivity [73]). Furthermore, social support from family members was found to buffer effects of maternal trauma exposure on child internalizing problems (at low levels of trauma exposure [71]) and externalizing problems [72]. Given these buffering effects of parental social support on adverse parenting outcomes and child risk of mental illness, we consider strengthening the parental social support network an important intervention target.

### Parenting stress

Parents with PTSD generally experience the parenting role as more stressful and less satisfying than parents without PTSD [56, 59]. Parenting stress is not only distressing for the parent, but can also have harmful effects on child wellbeing. Parenting stress has been found to mediate between parental trauma exposure or PTSD and child emotion regulation, internalizing and externalizing symptoms [63, 74, 75]. Furthermore, there is evidence that parenting stress can contribute to maintenance of PTSD symptoms [76]. Reduction of parenting stress is not a direct target of KopOpOuders-PTSD, but we hypothesize that parenting stress may be reduced as a result of changes parents are encouraged to make in the intervention (e.g., learning effective boundary setting skills, reducing guilt surrounding the need for alone time and relaxation, sharing childcare responsibilities with the social support network). Given the importance of parenting stress in the association between parental PTSD and child and parent wellbeing, we consider it useful to test whether participating in KopOpOuders-PTSD reduces parenting stress.

Concluding, preventive parenting intervention is a promising avenue towards prevention of mental health problems in children of parents with PTSD. Enhancing adaptive parenting behavior, promoting perceived parenting competence, and strengthening parents’ social support network can have positive effects on both parents with PTSD and their children. In this article we describe the development of KopOpOuders-PTSD, a blended care preventive parenting intervention for parents with PTSD, and explain how the effectiveness of this intervention will be tested.

### Aims of this study

#### Primary objective

To test the effect of KopOpOuders-PTSD on macro- and micro-level parenting behavior by answering the following research questions:

1. What is the effect of KopOpOuders-PTSD on macro-level parenting behavior?

H1. We hypothesize that parenting behavior at posttest will be more adaptive in the intervention condition than in the control condition. This effect is hypothesized to remain stable at follow-up.

2. What is the effect of KopOpOuders-PTSD on micro-level parenting behavior?

H2. We hypothesize that micro-level parenting behavior will be significantly more adaptive at posttest in the intervention condition than in the control condition.

#### Secondary objectives

To test the effect of KopOpOuders-PTSD on secondary macro-level outcomes by answering the following research questions:

3. What is the effect of KopOpOuders-PTSD on perceived parenting competence?

H3. We hypothesize that perceived parenting competence will be significantly greater at posttest in the intervention condition than in the control condition.

4. What is the effect of KopOpOuders-PTSD on parental social support?

H4. We hypothesize that parental social support will be significantly greater at posttest in the intervention condition than in the control condition.

5. What is the effect of KopOpOuders-PTSD on parenting stress?

H5. We hypothesize that parenting stress will be significantly lower at posttest in the intervention condition than in the control condition.

6. What is the effect of KopOpOuders-PTSD on child overall psychological problems?

H6. We hypothesize that child overall psychological problems will be significantly lower at posttest in the intervention condition than in the control condition.

7. What is the effect of KopOpOuders-PTSD on child PTSD symptoms (in children who have also experienced trauma)?

H7. We hypothesize that for children who have experienced trauma, PTSD symptoms will be significantly lower at posttest in the intervention condition than in the control condition.

These effects are all hypothesized to remain stable at follow-up.

To test for moderation effects of parental PTSD symptoms by intervention condition on macro-level outcomes by answering the following research question:

8. Are intervention effects on macro-level endpoints at posttest moderated by parental PTSD symptoms?

H8. We hypothesize that intervention effects will be smaller for parents who have higher PTSD symptoms at pretest.

## Method

### Intervention

#### The online platform

KopOpOuders-PTSD consists of five weekly online modules and three face-to-face sessions with a professional (see Additional file 2 for a summary of the modules and sessions). The online modules can be accessed through an online platform. Each module follows the same structure, with components such as reflection on the past week, videos of interviews with other parents with mental illness, and a homework exercise. Modules take ca. 30–45 minutes each to complete, and homework exercises ca. 20 minutes. In addition to the online modules, the platform also contains a ‘library’ with additional information about PTSD and other mental illnesses, how to explain these to children, their potential impact on children, and risk and protective factors. Finally, the platform contains a ‘self-care hub’ with content such as relaxation exercises and positive writing exercises [38].

#### Adapting KopOpOuders self-help into KopOpOuders-PTSD

KopOpOuders Self-Help was adapted for the PTSD population in three ways: integration of perspectives and needs of parents with PTSD; expansion from online to blended care; and addition of PTSD-specific content.

#### Integrating parents’ PTSD-specific needs

To ensure that the form and content of KopOpOuders-PTSD matches the wishes and needs of the target group, we adopted a co-creation approach, in which we collaborate with an advisory board of parents with PTSD and their partners. The advisory board fluctuated in size and composition throughout the development of the intervention, ranging from three to six members. Input from advisory board members was obtained in focus group-like meetings from the start of intervention development and will continue to the end of the study. As far as possible within the small group, we strived to ensure diversity in gender, ethnic backgrounds, (dis) abilities and family compositions. Whereas the current advisory board is diverse in most aspects, some groups (e.g., single-parent families, LGBTQIA+ families, families with infant children) were not sufficiently represented. Apart from diversity, we also strive for inclusivity in the advisory board. This means that we endeavor to make all members feel equally valued, respected, and safe to be themselves; that we compensate members for their expertise; and that we continue to acknowledge and challenge our own privilege and biases.

The knowledge gained from the advisory board was supplemented with results from a mixed methods study we conducted among parents with PTSD. In this study, parents were asked about their wishes and needs for parenting support and their opinions on aspects of the intervention protocol. Table 1 presents an overview of adaptations to the study protocol and intervention design based on input from the advisory board and mixed methods study.

**Table 1:** Intervention and study design decisions based on input from advisory board and mixed methods study

**Source**	**Intervention: Online Modules**
Advisory board	Advisory board members pointed out module content was sometimes too confrontational, which could be improved through small changes. E.g., green and red markers to denote parenting strengths and difficulties, were replaced by neutral-colored markers labeled ‘this is something I do well’ and ‘this is something I find challenging and want to learn more about’.
Advisory board, Mixed methods study	To every online module, extra texts were added which provided a PTSD-specific perspective on the module topic. The content of these texts was based on the personal experiences of advisory board members and mixed methods study participants.
Advisory board	Advisory board members suggested adding more concrete ‘tips and tricks’ to the extra texts. We based these partially on tips that the board members and their families use themselves.
Advisory board	Advisory board members emphasized the importance of leaving the house, exercising, and social connection for relaxation. We therefore added more tips about this, balancing out the tips which had been more focused on being alone and at home (e.g., yoga and meditation).
	**Intervention: In-Person Sessions**
Advisory board, Mixed methods study	Opinions were mixed on whether to carry out sessions one-on-one or in a group with multiple parents. Most parents expressed concerns about privacy and trust in group settings, while also seeing value in the recognition that could be gained from hearing other parents’ experiences. In the end, we decided on one-on-one sessions. The element of recognition was promoted by presenting the extra texts in the online modules as conversations between fictionalized parents with PTSD sharing their struggles and advice with each other.
Advisory board	Advisory board members recommended “do’s and don’ts” for the professionals leading the sessions (e.g., being aware of potential mistrust in institutions, especially in veteran parents; parents appreciate it when professionals are well-prepared, having an open and accepting attitude, and refraining from assumptions and judgment). These were integrated in the protocol and training for professionals.
Advisory board, Mixed methods study	The following themes, which advisory board members and mixed methods study participants raised as important, were integrated in the protocol for the in-person sessions: - On the one hand, parents experience shame and guilt towards their children/family for having PTSD; on the other hand, they also experience shame and guilt about recovering from PTSD, perceiving themselves as “too self-absorbed” or “not available enough” (e.g., being too tired after therapy sessions to participate in family activities). Recognizing and reducing these conflicting feelings is important; - Practical tips for communication with family when feeling overwhelmed or being triggered; - Recognizing and reducing overprotectiveness and overcontrol in the home and family setting.
Advisory board	All advisory board members emphasized the importance of involving the partner. We therefore adapted the protocol of the final session, so that parents can bring their partner (or other significant person) to discuss their progress and how the partner can support them.
	**Ecological Momentary Assessment**
Advisory board	The assessment schedule was changed, to make it less demanding and more flexible, due to concerns from the advisory board about the difficulty of completing EMA assessments during busy times with their child.
Advisory board	Items about sleep quality and overall distress level were added, because advisory board members pointed out these aspects had a large impact on how their day was going to be.
	**Recruitment Materials**
Advisory board	The recruitment flyer was changed to be simpler and less text-heavy; advisory board members remarked that the setting in which the flyer would be encountered (the treatment center) is an environment in which they often feel tense and would have insufficient focus to read large amounts of text.

#### Expansion to blended care

The three minimal contact moments were replaced by three 90-minute in-person sessions with a preventive mental health professional. To uphold the emphasis on parental autonomy and self-direction while instituting more extensive contact with a professional, the role of the professional is that of ‘facilitator’ rather than ‘leader’. All professionals receive a 12-hour training, and sessions are carried out according to a manual to ensure consistency. Whereas the content of the online modules remains largely the same as in the original KopOpOuders Self-Help-intervention (and thus applicable to a wide range of mental health and addiction problems), the in-person sessions have a more in-depth and PTSD-specific focus. In the sessions, parents receive psychoeducation about PTSD and parenting, are encouraged to ask for individualized guidance, and get the opportunity to practice newly learned skills. In-person sessions are carried out one-on-one, but parents are encouraged to bring a partner or other significant person in the final session.

#### Addition of PTSD-specific content

In KopOpOuders-PTSD, the transdiagnostic (i.e., not specific to any one diagnosis) protective factors of KopOpOuders Self-Help are supplemented with PTSD-specific factors. This is done in two ways. Firstly, intervention themes are approached from a PTSD-specific perspective in in-person sessions. For example, one of the themes in KopOpOuders Self-Help is ‘talking to your child about your illness’. After parents learn about this in a more general way in the online module, the following in-person session addresses PTSD-specific aspects, including constructive and age-appropriate ways to talk to children about PTSD and trauma. Secondly, PTSD-specific content is added to the online intervention, which parents can refer to according to their own needs. The content is presented in the form of written conversations between fictional parents with PTSD, to increase relatability and recognition. The written conversations cover topics such as recognizing and dealing with triggers in the family setting, and the impact of PTSD-related avoidance and dissociation on children.

### Setting

Inclusion of participants started June 1st, 2022, and is expected to finish December 31st, 2023. Participants are outpatient clients in treatment for PTSD at Arkin Mental Health Care, a Dutch mental health care provider based mainly in the city Amsterdam. Clients from several Arkin Mental Health Care departments can participate: Sinai Centrum (for clients with primary diagnosis of PTSD), Jellinek (for clients with primary diagnosis of substance use disorders), Arkin BasisGGZ (for clients receiving low-intensity basic mental health care) or NPI (for clients with main diagnosis of personality disorders). PTSD treatment at these centers, which we refer to as Treatment as Usual (TAU), consists of Eye Movement Desensitization and Reprocessing, Imaginary Exposure, and/or Imagery Rescripting Therapy.

### Study design

This study uses a two-arm randomized controlled trial (RCT) design: the intervention condition receives KopOpOuders-PTSD in addition to TAU and is compared to a control condition which only receives TAU. This design allows us to test whether the effect of KopOpOuders-PTSD in addition to TAU is superior to that of TAU only. The randomization ratio is 1:1. Block randomization (2:4:6) is used, stratified by the Arkin Mental Health Care department (Sinai Centrum or other; clients of Sinai Centrum have PTSD as their primary diagnosis, whereas clients of other departments typically do not) to ensure even distribution across conditions. Double-blind randomization is not possible, because participants and prevention professionals providing in-person sessions know automatically whether the participant is receiving the intervention. Therefore, we apply single blinding, in which the researcher overseeing the assessments and analyzing the results is blind to the condition. Randomization is done by a research assistant who is not involved in the assessments. Randomization outcomes are only accessible to this research assistant. Participants are instructed not to share their randomization outcome with the researcher overseeing their assessments, and professionals carrying out the intervention are instructed not to discuss any identifying details of intervention participants with the researcher. Unblinding is possible if needed in case of (serious) adverse events, and can be done by the primary investigator, who is not involved in assessments. To ensure parenting support is not withheld due to participation in this study, control condition participants are invited to complete the online KopOpOuders-PTSD modules after their participation and will receive information about the parenting support options at Arkin Mental Health Care.

#### Measurement levels

In this study, we distinguish between ‘macro-level’ and ‘micro-level’ outcomes. With macro-level, we refer to longer-term retrospective reports on behaviors, attitudes, symptoms, etc. By contrast, we use the term micro-level to refer to moment-to-moment dynamics of behavior and interactions in daily life. Whereas the macro-level assessments in this study are useful to detect overall changes in parenting and mental health outcomes from pretest to posttest, they may not be able to fully capture the dynamic nature of parenting in daily life. This is especially relevant in PTSD, where symptoms can fluctuate strongly throughout the day, and PTSD-related memory impairments may bias retrospective assessments [77]. We therefore supplement macro-level assessments with micro-level assessments using ecological momentary assessment (EMA), a research method in which participants systematically and repeatedly report on current or recent behavior, emotions and/or thoughts [78]. EMA can provide rich, ecologically valid information on moment-to-moment parenting dynamics [77–79].

#### Macro-level assessments

All outcomes in this study are measured at the macro-level using questionnaires at three measurement points: pretest (week 0), posttest (week 10), and follow-up (week 22). All questionnaires are completed by participants using the online platform Castor Electronic Data Capture [80].

#### Micro-level assessments

Micro-level assessment of parenting and PTSD symptoms is carried out through EMA at pretest and posttest. Participation in the EMA component of the study is optional. Participants who choose to take part can do so by installing the app ‘Ethica Data’ [81] on their smartphone. The Ethica Data-app sends a brief EMA-questionnaire at three moments per day: at the end of the morning (randomly between 11:00–12:00), afternoon (randomly between 16:00–17:00, and evening (randomly between 21:00–22:00). If a questionnaire is missed, a reminder signal is sent after 60 minutes. Questionnaires are locked after 120 minutes to preserve the ‘real-time’ effect and prevent accumulation of questionnaires.

### Participants

#### Sample size

We assumed a medium effect size for the primary macro-level outcome (parenting behavior: Alabama Parenting Questionnaire), *d* = 0.55, based on RCTs with similar designs, interventions and populations as the current study, and with the same primary outcome [21, 82]. With a power of 0.80 and significance level of *p* = .05, the minimum *n* is 53 per condition. From previous experience with intervention studies in adults with PTSD, we expect 20–25% dropout. We therefore corrected with 25% oversampling, resulting in 53/0.75 = *n* = 71 per condition, total *N* = 142.

For the primary micro-level outcome (parenting behavior: Short Parenting Scale for EMA), power was calculated using PowerAnalysisIL [83] using our best estimation of relevant parameters. Based on existing EMA literature [84], we assume participants will respond to 70% of the 42 EMA prompts, resulting in 29 complete data points per participant. Assuming an autocorrelation between measurement occasions of *r =* 0.30 and a medium effect size, minimum sufficient power to detect intervention effects would be reached if *n =* 32 (16 per condition) agreed to take part in the EMA component of the study.

#### Inclusion and exclusion criteria

Inclusion criteria for participation are: current DSM-5 diagnosis of PTSD; receiving PTSD treatment of at least three sessions at one of the participating Arkin Mental Health departments; and having parenting responsibilities for at least one child aged 4–17 (biological or legal relationship not required). Exclusion criteria are: urgent care needs which impede participation or (imminent) crisis (e.g., current psychosis, substance detoxification, active suicidality); no contact with children; receiving another parenting intervention during the participation period; severe psychological problems or intellectual disability in children (diagnosis of oppositional-defiant disorder, conduct disorder, current psychosis, personality disorder, or IQ < 50); and inability to participate in the intervention and/or assessments (e.g., unable to speak or read Dutch).

These criteria are similar to inclusion criteria for the original KopOpOuders Self-Help-intervention, but have been adapted to the goals and population of the current study (i.e., the first two inclusion criteria replace the more general KopOpOuders Self-Help criterion that parents should have some form of mental or substance abuse disorder) and on evaluation of KopOpOuders Self-Help (i.e., the age range of children was narrowed from 1 to 17 to 4–17, because intervention content is less applicable to younger children [38]. If a participant’s situation changes so that urgent care needs or severe family problems warranting intervention arise during study participation, their participation will be discontinued and their main practitioner will ensure they receive the appropriate support.

#### Recruitment

Participants will be recruited among clients of the abovementioned Arkin Mental Health Care departments. Practitioners will ask clients who meet inclusion criteria whether they agree to be approached for this study. Furthermore, continuous recruitment will take place at client intake. Part of the standard intake procedure is the ‘Child Check’, in which the client is asked whether they have parenting responsibilities, how old their children are, and whether their mental health problems affect their children. If during the Child Check a client who will receive PTSD treatment reports having children aged 4–17, they will be asked whether they agree to be approached for the study. Recruitment flyers and posters with contact information of the research team will also be placed in waiting rooms.

### Procedure

#### Pre-inclusion

When a client consents to being approached after receiving information from their practitioner, a research team member will call them to provide information about the study objectives and procedure and answer questions. If the client is interested in participation, the research team member will screen them for inclusion and exclusion criteria and record the result in a screening log.

The start of trauma therapy can bring about temporarily heightened emotional strain and risk of deregulation. During this period, there is a somewhat higher risk of exceeding the window of tolerance resulting in hyperarousal (e.g., increased intrusive symptoms) and/or hypoarousal (e.g., increased avoidance). In hyper- and hypoarousal states, affect tolerance and learning ability are temporarily decreased [85]. To prevent adverse emotional reactions and dropout, participants can therefore only start participating in the study after at least three sessions of PTSD treatment. Starting sooner is only possible if both client and practitioner consider this suitable and beneficial.

#### Inclusion, pretest, and randomization

The participant meets with a research team member for an inclusion, pretest, and randomization appointment, either in person at the treatment location or by video call. The participant receives and reads the participant information letter beforehand. First, the researcher answers any questions the participant might still have. The participant then signs the informed consent form. Next, the participant completes the pretest questionnaires online on a computer or tablet. The researcher remains in the room (or on the video call) to provide help if needed. If a participant has multiple children aged 4–17, they are instructed to report on the child they have most concerns about (the ‘index child’).

Participants who agree to take part in the EMA-component then receive instruction on how to install and use the EMA app, Ethica Data [81], on their smartphone. If a participant does not have a smartphone, they can borrow one from the research team. Participants also receive a written copy of the instruction including tips for troubleshooting the app if needed.

Finally, the participant is randomized to the intervention or control condition using the Castor Electronic Data Capture randomization module [80]. Participants are directly informed of their outcome. If a participant in randomized to the intervention condition does not have a computer, laptop or tablet at home, they will be instructed on how they can use computer rooms at their treatment location to complete the online modules.

#### Week 0: first EMA-period

The first EMA-period starts the day after pretest and runs for seven days. The app stops sending questionnaires automatically after the seventh day, but should remain installed for the second EMA-period.

#### Week 1–9: intervention period and/or continuation of TAU

Eight days after pretest, participants in the intervention condition receive an e-mail giving them access to the online intervention platform. A prevention professional contacts the participant to plan the in-person sessions. The five online modules and three in-person sessions are meant to be completed at a pace of one per week. The intervention period runs for nine weeks, giving participants extra time in case there is a week in which they do not manage to complete a module or session. For all participants, TAU will continue as normal during and following participation in this study.

#### Week 10: posttest

In week 10, all participants are contacted by a research assistant to plan the posttest assessment. To improve retention rates, each participant is contacted several times by phone and e-mail if the initial contact attempt is unsuccessful. Depending on their preference, participants can complete the questionnaires with support from a researcher on video call or at their treatment location.

#### Week 10: second EMA-period

The day after posttest, the second EMA-period starts. Measures and procedure are identical to the first EMA-period.

#### Week 22: follow-up

**Fig. 1:**
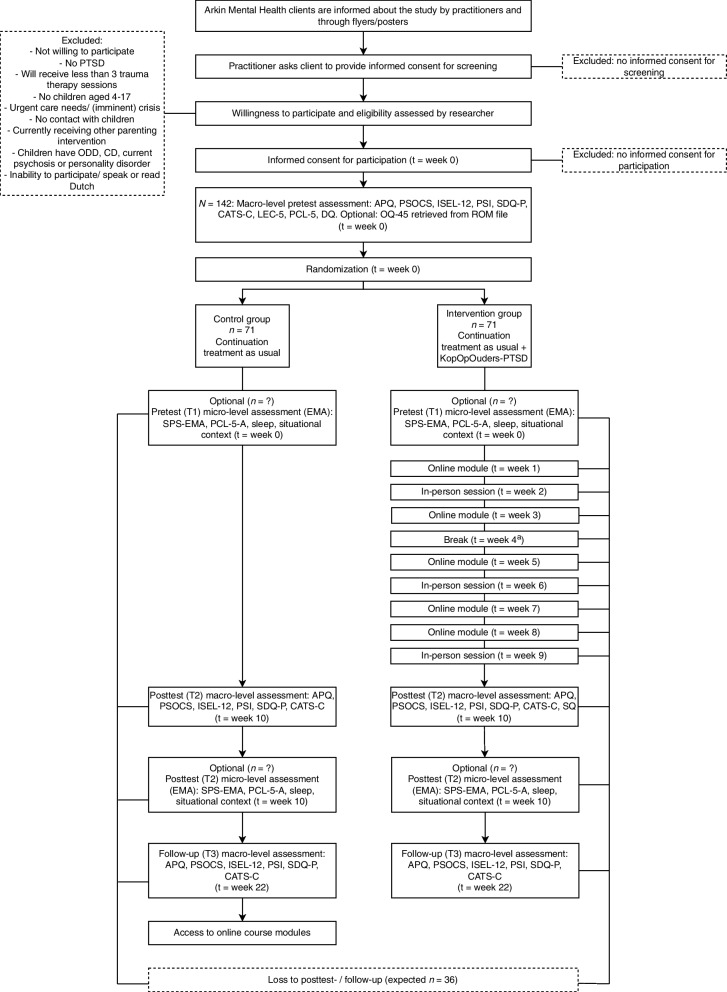
Flowchart of Study Procedure. Note. ^a^ The intervention period has a one-week margin in case there is a week in which the participant cannot participate (“Break”); in the figure, the break is in week 4, but this may vary according to participant preference

In week 22, all participants are contacted by a research assistant to plan an appointment for the follow-up assessment. The procedure is identical to that at posttest. After completing the questionnaires, control condition participants receive an e-mail inviting them to the online KopOpOuders-PTSD modules and highlighting parenting support options at Arkin. Figure 1 depicts an overview of the procedure.

#### Adherence and compliance monitoring

To accurately interpret intervention effects, documenting treatment adherence and compliance in participants and prevention professionals is important. Participants’ adherence is monitored in three ways: completion of online modules and use of PTSD-specific content in the online library (monitored automatically by the intervention platform software), and attendance of in-person sessions (recorded by the prevention professional leading the sessions). In prevention professionals, implementation fidelity is promoted and monitored through mandatory training and monthly intervision sessions with the research team. In each intervision session, a research team member will capture factors affecting intervention fidelity through implementation logs. These implementation logs will be used to identify where guidance and feedback is needed in the intervision sessions. Furthermore, they will be thematically analyzed and described in an implementation report after completion of the study.

### Instruments

#### Primary outcomes

##### Macro-level: parenting behavior

Parenting behavior is measured using the Dutch translation of the caregiver-reported Alabama Parenting Questionnaire (APQ [86, 87]). The APQ is a 42-item questionnaire with five subscales: positive involvement with children, supervision/monitoring, use of positive discipline techniques, consistency in discipline techniques, and use of corporal punishment. Items describe parenting behaviors and situations (e.g., ‘You don’t tell your child where you are going’), which are scored for frequency on a five-point Likert scale ranging from ‘never’ (1) to ‘always’ (5). APQ total scores range from 42 to 210. However, subscales are often analyzed separately, with higher scores reflecting higher parenting skills on the ‘positive involvement’ and ‘positive discipline techniques’ subscales, and lower parenting skills on the other three subscales. In this study, we will use the total score as the primary outcome for the main analysis (research question 1) and perform additional analyses on the separate subscales. The APQ has good psychometric properties, with adequate to good internal consistency [88, 89] good test-retest reliability [90] and good construct validity [88, 89].

##### Micro-level: real-time parenting behavior

When participants report being currently or recently in contact with the index child in the EMA questionnaire, parenting behavior is assessed using eight adapted items from the Parenting Behavior Inventory (PBI [91]). The Dutch translation of the PBI was obtained from Visser and colleagues [92]. The PBI contains 20 items which comprise two independent subscales: hostile/coercive and supportive/engaged. Items describe parenting behaviors, which parents rate for frequency on a seven-point Likert scale ranging from ‘Never’ (1) to ‘Always’ (7). The PBI has good psychometric properties, with excellent internal consistency for both independent subscales, adequate to good content validity, and good test-retest reliability [91]. We have selected eight PBI items, of which four from each subscale. The wording of these items has been changed slightly to fit with the EMA format (e.g., ‘I say mean things to my child that could make them feel bad’ becomes ‘This morning… I have said mean things to my child that could make them feel bad’). We have also changed the response scale to a three-point Likert scale: ‘Not true’ (1), ‘Somewhat true’ (2), ‘Certainly true’ (3). Scores on each of the two subscales range from 4 to 12. The total score of the two subscales combined ranges from 8 to 24. This total score will be used to test intervention effects (research question 2). For this study, we will call this questionnaire the ‘Short Parenting Scale for EMA’ (SPS-EMA). Because this novel adaptation of the PBI has not previously been used in EMA, we tested the SPS-EMA in a small-scale pilot study. We found the items to have sufficient frequency and variability for use with EMA.

#### Secondary endpoints

##### Macro-level: perceived parenting competence

Perceived parenting competence is measured using the Dutch translation of the Parenting Sense of Competence Scale (PSOCS [93]). The PSOCS is a 16-item questionnaire comprising two subscales: satisfaction and efficacy. We will use the total score for the main analysis (research question 3) and perform additional analyses on the two subscales. Items are posed as statements (e.g., ‘Being a parent makes me tense and anxious’), which are scored on a six-point Likert scale ranging from ‘strongly disagree’ (1) to ‘strongly agree (6). PSOCS total scores range from 16 to 96. The PSOCS has adequate to excellent internal consistency, good test-retest reliability, and good construct validity [93–95].

##### Macro-level: social support

Parent social support is measured using the abbreviated version of the Interpersonal Social Support Evaluation List (ISEL-12 [96]). The ISEL-12 is a 12-item questionnaire comprising three subscales: appraisal support (availability of someone to talk to about problems), belonging support (availability of someone to do things together with) and tangible support (availability of someone to help with practical matters). We will use the total score for our main analysis (research question 4). Items describe statements (e.g., ‘I feel that there is no one I can share my most private worries and fears with’), which the participant rates for agreement on a four-point scale ranging from ‘Definitely false’ (1) to ‘Definitely true’ (4). ISEL-12 total scores range from 12 to 48, and will be coded in such a way that higher scores indicate more support. The ISEL-12 total score has good psychometric properties, with adequate to good internal consistency, adequate test-retest reliability, and good construct validity [96, 97]. We translated the ISEL-12 to Dutch using the back-translation method. We also added two self-made items reflecting aspects of social support we were interested in in light of our intervention: ‘There is someone who could look after my child(ren) for a day if I were not doing well’ (tangible support) and ‘If I were worried about the impact of my problems on my child, there would be someone I could talk to about it’ (appraisal support). These will be analyzed descriptively.

##### Macro-level: parenting stress

Parenting stress is measured using parent-report on an abbreviated Dutch version of the Parenting Stress Index (PSI [98, 99]). This abbreviated version is a 25-item questionnaire measuring stress related to childrearing and the parental role. Items are statements (e.g. ‘There are some things my child does that really bother me a lot’) which parents rate for agreement using a 6-point Likert scale ranging from ‘Totally disagree’ (1) to ‘Totally agree’ (6). We will use the total score as our analysis outcome (research question 5). The Dutch version of the PSI has acceptable validity [99].

##### Macro-level: child overall psychological problems

Child overall psychological problems are measured using parent-report on the Dutch translation of the Strengths and Difficulties Questionnaire-Parent Report (SDQ-P [100, 101]). The SDQ-P is a 25-item questionnaire suitable for parent-report on emotional and behavioral symptoms of children aged 4–17. The SDQ-P comprises five subscales with five items each: hyperactivity/attention deficit, emotional problems, behavioral problems, peer relationship problems, and prosocial behavior. SDQ-P items describe characteristics or behaviors (e.g., ‘Many worries, often seems worried’), for which parents rate to which extent these are true for their index child on a three-point Likert scale ranging from ‘Not true’ (0) to ‘Certainly true’ (2). We will use the ‘general difficulties’ score as our analysis outcome (research question 6), which aggregates all subscales excluding prosocial behavior (thus comprising 20 items). We will perform additional analyses on the separate subscales. The ‘general difficulties’ score ranges from 0 to 40. The SDQ-P has good psychometric properties, with adequate internal consistency and test-retest reliability and good construct validity [102].

##### Macro-level: child PTSD symptoms

Child PTSD symptoms are measured using parent-report on the Dutch translation of the Child and Adolescent Trauma Screener-Caregiver Report (CATS-C [103, 104]). The CATS-C has two versions; one for children aged 3–6 and one for children aged 7–17. Both versions start with 15 yes/no items about child exposure to DSM-5 A-criterion traumatic events (e.g., ‘Serious natural disaster like a flood, tornado, hurricane, earthquake, or fire’). We have added one item (‘Learning from me or someone else about the traumatic experience(s) I have had’), because this potentially traumatic experience was not included in the original questionnaire. Furthermore, we have added an item to assess whether the parent and child were co-exposed to the same trauma (e.g., being in an accident together, shared exposure to domestic violence). This item states: ‘Was your child present during a traumatic event you experienced? For example, did you experience it together, or did your child witness it happening to you?’

If the parent reports their child has been exposed to at least one traumatic event, the questionnaire continues with 16 (age 3–6) or 20 (age 7–17) items about PTSD symptoms (e.g., ‘Being overly alert or on guard’). Data on PTSD symptoms are thus only collected if the child has experienced at least one A-criterion traumatic event. PTSD symptom items are rated for frequency in the past two weeks on a four-point Likert scale ranging from ‘Never’ (0) to ‘Almost always’ (3). The CATS-C also contains five yes/no items about interference with life domains (e.g., ‘Getting along with others’). The PTSD symptom items are divided into four subscales corresponding with DSM-5 PTSD symptom clusters. Total symptom scores range from 0 to 48 (age 3–6) or 0–60 (age 7–17). In this study, we use the total symptom scores as our analysis outcome (research question 7). We will perform additional analyses on the separate subscales. Scores on trauma exposure items will be reported descriptively. The PTSD symptom measure of the CATS-C has good psychometric properties, with good to excellent internal consistency and good construct validity [103]. As of yet, test-retest reliability has only been assessed for the self-report version of the CATS, which shows adequate test-retest reliability [105].

### Background and moderator variables

#### Macro-level: trauma type/timing

Lifetime trauma exposure will be measured at pretest using the Dutch translation of the Life Events Questionnaire for DSM-5 (LEC-5 [106, 107]). The LEC-5 comprises 17 items which describe DSM-5 A-criterion traumatic events (e.g., ‘Fire or explosion’). Participants note for each event on a scale with six options whether they were directly or indirectly (i.e., as a witness, learning about it happening to someone close to them, or as part of their job) exposed to it. In case of multiple trauma exposures, the participant is asked to indicate which event bothers them the most (the ‘index trauma’). Psychometric properties for the LEC-5 have not yet been established, but the highly similar previous version (LEC-IV) has good psychometric properties, with adequate test-retest reliability and good construct validity [108].

#### Macro-level: PTSD symptoms

PTSD symptoms will be measured at pretest using the Dutch translation of the PTSD Checklist for DSM-5 (PCL-5 [107, 109]). The PCL-5 comprises 20 items describing PTSD symptoms in relation to the index trauma (e.g., ‘Becoming upset when something reminded you of the stressful experience’), which are rated for past-month frequency on a five-point Likert scale ranging from ‘Not at all’ (0) to ‘Extremely’ (4). The PCL-5 comprises four subscales corresponding to DSM-5 PTSD symptom clusters (intrusion, avoidance, negative alterations in mood and cognitions, and hyperarousal). We will use the PCL-5 total score as a moderator variable (research question 8). PCL-5 total scores range from 0 to 80. The PCL-5 has good to excellent psychometric properties, with excellent internal consistency, good test-retest reliability and good construct validity [109].

#### Macro-level: psychosocial functioning

Among the most common comorbid disorders with PTSD are major depressive disorder, anxiety disorders and substance abuse disorders. Problems in life domains such as relationships and professional functioning are also common in PTSD [83]. We therefore report descriptively on general psychosocial functioning in the sample. This is measured as part of routine outcome monitoring at Arkin Mental Health Care using the Outcome Questionnaire-45 (OQ-45 [110]). We will use the closest measurement before pretest. The OQ-45 is a commonly used questionnaire for measuring clinical outcomes. It comprises 45 items, which are divided into three subscales: symptom distress (25 items, measures symptoms of depression, anxiety and substance abuse), interpersonal relations (11 items, measures relational functioning) and social role (9 items, measures functioning in school, work and/or leisure). Items describe symptoms/situations (e.g., ‘I feel no interest in things’), which participants rate for past-week frequency on a 5-point Likert scale ranging from ‘Never’ (0) to ‘Always’ (4). We will report the OQ-45 total score, which ranges from 0 to 180, and subscale scores, which range from 0 to 100 (symptom distress), 0–44 (interpersonal relations), and 0–36 (social role). The OQ-45 has good psychometric properties, with good test-retest reliability, adequate to excellent internal consistency, and good construct validity [110].

#### Macro-level: demographic information

To provide information on the demographic background of the sample, participants complete a short demographic questionnaire (DQ) at pretest with items about characteristics such as age, gender, employment status, family composition, and number of children.

#### Macro-level: intervention adherence and satisfaction

For participants in the intervention condition, intervention adherence (completion of online modules; attendance of in-person sessions; and use of PTSD-specific content in the online library) will be reported descriptively. Furthermore, these participants complete a short satisfaction questionnaire (SQ) at posttest. The SQ contains closed-ended and open-ended questions on aspects of the intervention, and invites participants to provide suggestions for improvement. Results will be reported descriptively in the publication of the current study and will be used for future improvement of the intervention.

#### Micro-level: parental PTSD symptoms

PTSD symptoms are assessed using EMA with an abbreviated version of the PTSD Checklist for DSM-5 (PCL-5-A). This version contains the 10 PCL-5 items with the strongest factor loading for their respective symptom cluster. It was created for and used successfully in previous EMA research [111, 112]. The PCL-5-A contains three items for intrusion, two for avoidance, two for negative cognitions and mood, and three for hyperarousal. Participants are asked to report on their symptoms during the current part of the day using the same five-point Likert scale as in the regular PCL-5 (‘Not at all (0) to Extremely (4). Total scores range from 0 to 40.

#### Micro-level: sleep quality

Sleep quality is assessed in the first EMA assessment of each day, using a single item (‘Last night, how would you rate your sleep quality overall?’) from the Pittsburgh Sleep Quality Index [113]. Sleep quality is rated from ‘Very good’ (0) to ‘Very bad’ (3). The normal instruction is ‘During the last month’; this is replaced with ‘Last night’ to fit the EMA format. This adaptation has been used successfully in previous EMA research [111].

#### Micro-level: situational context

To assess the situational context, each EMA questionnaire starts with two situation items (Where are you?; What are you doing?), answered using multiple choice.

### Data management

Data management is carried out according to a protocol to ensure security and quality. This data management protocol can be found on our Open Science Framework page [114].

### Safety reporting and monitoring

Research team members involved in assessments and contact with participants and prevention professionals carrying out the intervention sessions are instructed in the reporting procedure of (serious) adverse events. These must be reported to the primary investigator according to guidelines by the Central Committee for Research in Humans [115]. Engaging a data monitoring committee or auditor and performing interim analyses is considered not to be necessary due to the low-risk nature of the study.

### Analysis

Primary analyses will be conducted on the entire randomized sample (i.e., intention to treat). Extra analyses for comparison will be conducted on the per protocol/treatment completers sample. Participants in the intervention condition who have completed at least six out of eight intervention modules/sessions will be characterized as treatment completers. Participants who fail to complete the intervention, but do not choose to drop out of the study, will complete the posttest and follow-up assessments as normal. Hypothesis testing will be two-sided with an alpha level of .05. Missing data will be handled using multiple imputation (R package MICE or equivalent).

#### Macro-level: primary and secondary outcomes

Analyses of primary (research question 1) and secondary outcomes (research questions 3–7) on the macro-level will be carried out using generalized linear mixed modeling (LMM). For ratio scale endpoints or if distributions of endpoints are skewed, robust LMM is used. Separate models are run for each endpoint. Fixed factors are condition (intervention/control), time (pretest/posttest/follow-up), and the interaction effect of condition*time. Random intercepts are fitted at the participant level. The pretest value of the endpoint is included as a control variable. In a secondary sensitivity analysis, we will also include parental gender, parental age, child age, EMA participation (yes/no), trauma type (interpersonal/non-interpersonal), child exposure to trauma (no trauma exposure/co-exposed to parental trauma/exposed to trauma separate from the parental trauma), and number of comorbid psychiatric diagnoses of the parent as covariates.

#### Micro-level: primary endpoint

To test the effect of the intervention on micro-level parenting behavior (research question 2), we use a multilevel LMM in which assessments (up to 42: three per day, seven days at pretest, seven days at posttest) are nested within participants. Random intercepts are fitted at the participant level. Two dummy variables, one for measurement period (pretest/posttest) and one for condition (intervention/control) are added to the model. Differences between conditions at posttest are analyzed, while controlling for pretest scores. Because there are several assessment points per day, we control for average autocorrelation between assessment points. Finally, we control for time trends by including a variable reflecting the amount of time elapsed since inclusion.

#### Macro-level: moderation analysis

To test whether treatment outcomes on macro-level endpoints at posttest are moderated by parental PTSD symptoms at pretest (research question 8), we modify the models for macro-level endpoints by using only the posttest measure of each endpoint and including the moderator variable (PCL-5 total score). Hypothesis 8 is tested using the interaction effect of condition*PCL-5 total score on posttest measurements of the endpoint, while controlling for pretest measurements of the endpoint. A separate moderation model will be run for each endpoint.

#### Descriptive analyses

Descriptive statistics (for continuous variables: means, standard deviations, ranges, correlations; for categorical variables: frequencies, correlations) are reported for all primary and secondary outcomes and background variables. Descriptive findings regarding participants’ satisfaction with the intervention are summarized in text.

### Dissemination policy

The results of this study will be submitted for publication in an international peer-reviewed open access journal. The principal investigator will determine publication strategy. Results will also be communicated to the public through online newsletters. KopOpOuders-PTSD will be made available to other institutions within the Netherlands if it is found to be effective in this study. The online modules can be obtained under license from Trimbos Institute; the protocol for the face-to-face sessions will be made freely available to institutions when they acquire this license. A process evaluation report will be published to inform other professionals about the implementation process, potential challenges and solutions. Institutions wanting to implement KopOpOuders-PTSD can refer to this process evaluation report for implementation advice.

## Discussion

This article describes the development and RCT protocol of KopOpOuders-PTSD, a preventive blended care intervention for parents with PTSD, aimed at preventing child psychological problems by improving parenting outcomes. Intervention effects will be tested using macro-level (questionnaire) and micro-level (EMA) data. On the macro-level, we hypothesize that adaptive parenting behavior, perceived parenting competence and parental social support will be significantly greater and parenting stress significantly lower at posttest in the intervention condition than in the control condition. Furthermore, we hypothesize that child overall psychological problems and PTSD symptoms will be significantly lower at posttest in the intervention condition than in the control condition. Changes are hypothesized to remain stable at three-month follow-up. Intervention effects are hypothesized to be smaller for participants with higher PTSD symptoms at pretest. On the micro-level, we hypothesize that parenting behavior will be significantly more positive at posttest in the intervention condition than in the control condition.

Some limitations of this study protocol must be noted. Firstly, the study relies on parent-report. Validity of findings may benefit from an independent assessor (e.g., observations of parent-child interactions) and/or multiple informants (e.g., parent and child-report). In this study, this is not feasible because it would result in significantly higher participant burden as well as ethical concerns (i.e., involving children would be difficult because they may not yet be aware of their parent’s PTSD). Secondly, some parents who might benefit from parenting support are excluded from participation in this study, namely those with severe problems in the family (e.g., acute and severe psychological problems in the parent or child, family problems resulting in no contact with children) and those who do not understand Dutch. Due to its preventive nature, KopOpOuders-PTSD is insufficient for families with severe problems, and versions of the intervention in other languages than Dutch are not currently available. Providing interventions to non-Dutch speaking parents remains an important objective for the future, especially considering that some ethnic minority groups are at increased risk for developing PTSD [2].

Despite these limitations, this study also has several strengths. Firstly,, it is among the first to develop and test a PTSD-specific parenting intervention. Whereas several effective and beneficial interventions have catered to trauma-exposed parents and parents with other types of mental illness, KopOpOuders-PTSD is specifically tailored to parents with PTSD. This is important given the potentially detrimental effect of PTSD symptoms on intervention adherence and effectiveness [26–28] and the specific challenges parents with PTSD may face [30–33, 36]. The co-creation approach allows us to meet parents’ needs more closely by directly integrating their vision. Secondly, the blended care format of KopOpOuders-PTSD is innovative and provides a mix of flexibly usable online modules and individual in-person guidance. Finally, the use of EMA provides valuable information on how intervention effects may play out in real-life situations [77–79].

Concluding, this RCT will be the first to test the effectiveness of a PTSD-specific parenting intervention in addition to TAU. Results can contribute to clinical practice by demonstrating whether preventive parenting intervention can contribute to the wellbeing of parents with PTSD and their children.

## Supplementary Information

**Additional file 1: Appendix C.** Informed consent form for participants.

**Additional file 2.** KopOpOuders-PTSD session overview.

## Data Availability

Upon completion of the project, the following materials will be shared on the Open Science Framework repository [114]: metadata for the datasets generated by the study including data dictionaries, the study protocol, and statistical code used for analyses. Anonymized individual participant data (this includes data collected using questionnaires, EMA, and KopOpOuders user platform data for the intervention condition) will be made available upon request to the authors. Requests can be submitted through a data request form which will be made available on our Open Science Framework page upon completion of the project [114]. This data request form will require specification of the research questions, which must be in line with the informed consent conditions agreed to by participants (“scientific research concerning PTSD, trauma, parenting and/or child mental health”). All data will be fully anonymized and only shared for those participants who have consented to this prior to participation. Data will remain available for at least 15 years after completion of the project.

## Abbreviations

APQ: Alabama Parenting Questionnaire
CATS-C: Child and Adolescent Trauma Screener-Caregiver Report
DSM-5: Diagnostic and Statistical Manual of Mental Disorders- Fifth Edition
EMA: Ecological Momentary Assessment
ISEL-12: Interpersonal Social Support Evaluation List
LEC-5: Life Events Questionnaire for the Diagnostic and Statistical Manual of Mental Disorders- Fifth Edition
LGBTQIA+: Lesbian, Gay, Bisexual, Transgender, Queer/Questioning, Intersex, Asexual, +
LMM: Linear Mixed Modeling
MREC: Medical Research Ethics Committee
OQ-45: Outcome Questionnaire-45
PBI: Parenting Behavior Inventory
PCL-5: Post-Traumatic Stress Disorder Checklist For The Diagnostic And Statistical Manual Of Mental Disorders- Fifth Edition
PCL-5-A: Post-Traumatic Stress Disorder Checklist For The Diagnostic And Statistical Manual Of Mental Disorders- Fifth Edition – Abbreviated
PSI: Parenting Stress Index
PSOCS: Parenting Sense of Competence Scale
PTSD: Post-Traumatic Stress Disorder
RCT: Randomized Controlled Trial
SDQ-P: Strengths and Difficulties Questionnaire - Parent Report
SPS-EMA: Short Parenting Scale for Ecological Momentary Assessment
SQ: Satisfaction Questionnaire
TAU: Treatment As Usual
