# Supplementary material for: Study protocol: development and randomized controlled trial of a preventive blended care parenting intervention for parents with PTSD
Source: BMC Psychiatry. 2023 Feb 10;23:102. doi: 10.1186/s12888-023-04548-8 (PMC9921412; doi:10.1186/s12888-023-04548-8)
Supplement: Supplementary file 1 — Additional file 1: Appendix C. Informed consent form for participants. [file 12888_2023_4548_MOESM1_ESM.docx]

**
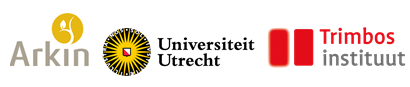
Appendix C: Informed consent form for particpants**

For **KopOpOuders-PTSD: Effectiviness of a course for parents with PTSD**

| - I have read the information letter. I was able to ask questions. My questions were answered sufficiently. I had enough time to decide whether I want to participate. - I know that participation is voluntary. I also know that I am free to decide not to participate at any time. Or to stop participating. I do not have to disclose why I want to stop. - I give the researcher permission to inform my main practitioner that I am participating in this study. - I give the researchers permission to collect and use my data from the questionnaires for this study. The researchers will only use these data to answer the research questions of this study. - I know that for monitoring of the study, some people can view all my data. These people are mentioned in the information letter. I give these people permission to view my data for monitoring. |
| --- |

**Can you please check yes or no in the table below? These options are not mandatory.**

| I want to participate in the part of the study with the smartphone-app, and I agree to collect and use the data from this app for **this study.** | Yes ☐ | No☐ |
| --- | --- | --- |
| I give permission to access my ROM and diagnostic information in my file, and use them for **this study.** | Yes ☐ | No☐ |
| I give permission to preserve the following, pseudonymized data after this study and share them for use in **other studies.** This can also be outside the EU: | | |
| Data from the questionnaires | Yes ☐ | No☐ |
| Data from the smartphone-app | Yes ☐ | No☐ |
| Data from my ROM and diagnoses | Yes ☐ | No☐ |
| Data about my use of the online modules, if I am randomized to option 1 | Yes ☐ | No☐ |
| I give permission to approach me for future research projects after this study is completed | Yes ☐ | No☐ |

**I want to participate in this study.**

My name is (participant): ………………………………..

Signature: ……………………… Date : __ / __ / __

I declare that I have fully informed this participant about the study. If during the study information becomes available which may influence the participant’s consent, then I will inform the participant in a timely fashion.

Name of researcher (or representative):……………………………….

Signature:……………………… Date: __ / __ / __

*The participant receives a complete information letter along with a signed version of the informed consent form.*
