# Supplementary material for: Study protocol: development and randomized controlled trial of a preventive blended care parenting intervention for parents with PTSD
Source: BMC Psychiatry. 2023 Feb 10;23:102. doi: 10.1186/s12888-023-04548-8 (PMC9921412; doi:10.1186/s12888-023-04548-8)
Supplement: Supplementary file 2 — Additional file 2. KopOpOuders-PTSD session overview. [file 12888_2023_4548_MOESM2_ESM.docx]

**Additional File 2. KopOpOuders-PTSD Session Overview**

***Online Module 1: Clarifying Family Situation and Needs.*** The main goal of the first online module is to create an optimal learning environment and clarify the participant’s goals. To this end, participants complete online exercises in which they describe their family situation, what problems they experience, what they missed in their own childhood, and what they want to learn or gain from the intervention. Next, participants reflect on their parenting, supported by a video of an interview with other parents, an animation, and an exercise in which participants rank a list of potential parenting challenges based on what they think are their own strengths and challenges. Recognizing and reducing negative cognitions about oneself as a parent is addressed in an interview between three parents with PTSD. The homework exercise is to practice ‘good enough parenting’, i.e. not having to be perfect, but making sure basic conditions such as safety, physical care, attention, predictability, and respect for child autonomy are guaranteed.

***Face-to-face session 1: PTSD, Parenting and Attitudes*.** The main goal of the first face-to-face session is for the professional and participant to get to know each other, and to establish a basis to work together. The session starts mutual introduction and clarification of expectations between the participant and professional. They then reflect on the first online module: does the participant have questions? Was there anything that stood out to them? What steps did they take following the module? What was challenging? Next, the professional provides education on common parenting challenges in people with PTSD and discusses with the participant whether these also apply to them. An infographic based on the four symptom clusters of PTSD is used for this. The professional discusses the learning goals the participant described in online module 1, and makes an assessment of what kind of (additional) support is needed. The session ends on a positive note: the participant is asked to say out loud and write down what makes them a good parent according to their child.

Handouts with homework exercises are given based on the participant’s learning goals:

- Establishing safety and calm at home and apologizing to your child (for parents who want to work on overreactivity/emotional outbursts).
- Cognitive restructuring exercises for reducing guilt and shame (for participants who want to work on negative thoughts and feelings about themselves as a parent).

***Online module 2: Effect of Parental PTSD on Child.*** The main goal of the second online module is promoting the protective factors parent-child interaction quality and child coping. The module starts with reflection on the ‘good enough parenting’ homework exercise and past week. Next, education about potential impacts of the parent’s mental health problems on the child and ways to apply adequate parenting strategies in mentally difficult times is provided through an interview video. After watching an animation about parentification, participants reflect on potential parentification of their child and whether their child has enough time for themselves. Lastly, parents learn about the potential roles children can play within the family when a parent has mental illness, and reflect on what role(s) their child plays. The homework exercise is for the parent to plan and execute a fun activity with their child. In the library, parents can find additional information about dealing with PTSD-related triggers, avoidance and overreactivity in the family setting.

Online module 3: Communication. The main goal of the third online module is promoting the protective factors parent-child interaction quality and child understanding of the parent’s illness. The session starts with reflection on the ‘fun activity’ homework exercise and the past week. Education about parent-child communication, active listening and talking to your child about PTSD is provided through an interview video. Parents reflect on how they (could) do this, and learn more on constructive communication about mental illness by watching an animation. They also complete an exercise about communication using ‘I-sentences’. In the library, parents can find additional information sheets with tips for talking to children of different ages about mental health and PTSD.

Face-to-face session 2: Talking about PTSD and Roles of the Child. The main goal of the second face-to-face session is promoting the protective factors parent-child interaction quality and child understanding of the parent’s illness. The session starts with the same reflection questions as face-to-face session 1. The professional provides an explanation on why it is important to talk to their child about having PTSD. The participant is asked to identify and write down at least one desired outcome of talking to their child about PTSD. If the parent does not feel ready to talk to their child, the professional and participant identify barriers together and make a plan to overcome these.

Next, the professional and parent reflect together on the roles of the child the participant has identified in Module 2. If there are concerns about the role the child takes on, the participant is asked to identify and write down concrete steps they will take to break this dynamic. The session ends on a positive note: the parent is asked to say out loud and write down what they have achieved in the intervention so far.

Handouts with reminders are given:

- A summary of “do’s and don’ts” for talking to your child about mental health.
- An overview of which roles children can play and why when a parent has mental illness.

Online module 4: Creating a Relaxed Family Situation. The main goal of the fourth online module is promoting the protective factors parent-child interaction quality and child resilience. The session starts with a reflection on the past week and the conversation with their child about PTSD. Education about setting boundaries on unwanted behavior and giving compliments to your child is provided through an interview video and a written interview between three parents with PTSD. Participants reflect on how they set boundaries, and learn about adequate ways to do so by watching an animation. Participants complete two exercises: one in which they describe three good characteristics of their child including concrete examples, and one in which they think about a situation in which their child shows unwanted behavior and how they would intervene. Because feeling positive emotions towards the child can be challenging and a source of guilt for parents with PTSD, recognition and reassurance about this is provided in a written interview between three parents with PTSD. The homework exercise is to give their child at least one compliment per day. In the library, participants can find additional information about recognizing and reducing overconcern, and about talking to children about romantic and sexual development (with specific attention to parents who are survivors of sexual abuse).

Online module 5: Social Support. The main goal of the fifth online module is promoting the protective factor informal social support for parent and child. The session starts with a reflection on the past week and on the compliments homework exercise. Participants reflect on their social support situation by watching an interview video in which parents discuss asking for support and situations in which they lacked support. Participants watch an animation about social networks and complete a ‘sociogram exercise’, in which they map their own social network and that of their child. Tips are provided about strengthening an existing social network, or establishing a social network if the participant does not have one. Participants draft an ‘emergency plan’: a preventive plan for when they need a time-out or are struggling and may not be able to care for their child. Finally, participants complete a ‘looking back and looking forward’ exercise, in which they reflect on what they have learned in the course, how they can apply this, and what they need more help with.

Face-to-Face Session 3: Looking to the Future. The main goal of the third face-to-face session is to integrate what participants have learned and identify potential needs for further support. In this session, the participant is encouraged to bring a partner or other significant person. The session starts with the same reflection questions as the other face-to-face sessions. If a partner or significant other is present, they are asked if they have noticed positive changes in the participant. If the participant did not bring someone, the professional answers this question.

The professional discusses the emergency plan with the participant and expands on it if needed. Practical requirements for execution of the plan are checked (e.g.: have people who are supposed to care for the child in an emergency been informed?). The objective is to create a plan for the period that the participant is in PTSD treatment and after, including both informal and (where needed) formal support.

The professional then gives the participant the opportunity to ask any questions they might still have, repeat previous exercises if desired, etc. Next, they look at the ‘looking back and looking forward’ exercise from online module 5 together to discuss if more professional support for the parent and/or child is needed and if yes, what steps should be taken. The session ends on a positive note: the participant and their partner are asked to say out loud and write down a wish and a positive affirmation for themselves and their child.
